# Complex Charcot Spine Reconstruction in a Young Paraplegic Patient: A Case Report

**DOI:** 10.7759/cureus.109595

**Published:** 2026-05-25

**Authors:** Tyler Krall, Jacob A Saunders, Isabel L Bauer, Kristin Nosova, Sananthan Sivakanthan

**Affiliations:** 1 Neurological Surgery, University of Arizona College of Medicine, Phoenix, USA; 2 Neurological Surgery, Banner - University Medical Center, Phoenix, USA

**Keywords:** case report, charcot spine, pseudomeningocele (pm), spinal cord injury, spinal fusion

## Abstract

Spinal Charcot arthropathy (SCA) is a rare, progressive, and destructive neurogenic joint disorder that develops in the setting of impaired sensory and autonomic innervation. Although once associated primarily with tabes dorsalis, spinal cord injury (SCI) is now the leading cause. The condition arises through a combination of autonomic dysregulation, bone resorption, and loss of proprioceptive feedback, resulting in progressive joint destruction and spinal instability. Diagnosis is often delayed, as symptoms such as deformity, instability, or autonomic dysreflexia (AD) may develop insidiously over the years.

We report the case of a 28-year-old female patient with paraplegia secondary to a chronic T5 SCI who presented with progressive thoracic deformity due to a severe Charcot joint at T12, associated with fracture-dislocation and a large pseudomeningocele. Despite complete thecal sac transection, her neurological function remained stable. Surgical management involved T5-pelvis posterior fusion with vertebrectomy, expandable cage placement, and a four-rod construct for maximal biomechanical stability. Intraoperative neuromonitoring was utilized throughout the procedure. The thecal sac was ligated to control cerebrospinal fluid (CSF) leakage. Postoperatively, the patient experienced transient hypotension consistent with autonomic dysfunction, but otherwise recovered well, achieving stable spinal alignment and resolution of her spinal deformity at three-month follow-up.

This case highlights several critical considerations in managing SCA, including the need for long-segment fixation extending to the pelvis to reduce recurrence, the advantages of multirod constructs for enhanced mechanical durability, and the importance of addressing CSF leaks from thecal sac injury. AD and hemodynamic instability must also be anticipated in this patient population. SCA should be suspected in paraplegic patients presenting with new deformity or instability, even at a young age. Early recognition and individualized surgical planning, including long-segment fusion, anterior column support, and definitive dural management, are essential to achieve durable stabilization and prevent recurrence. This case also highlights the importance of considering SCA among the differential diagnoses for progressive deformity in chronically paraplegic patients, while also recognizing alternative etiologies such as prior destabilizing surgery. This case contributes to the limited literature on SCA in young patients and offers practical insights for complex spinal reconstruction in neurologically impaired patients.

## Introduction

Charcot arthropathy is a progressive, destructive joint disorder that occurs in the setting of impaired sensory innervation [1]. The earliest documentation of neurogenic arthropathy involving the spine is attributed to Mitchell, who observed a patient with "caries of the spine" in conjunction with polyarticular lower extremity arthritis in 1831 [2]. Experimental work by Eloesser, in which posterior nerve roots were severed in cats, further elucidated the neurologic basis of Charcot joint pathophysiology [3]. Historically, spinal Charcot arthropathy (SCA) was most commonly associated with tabes dorsalis; however, the incidence has shifted in recent decades, with spinal cord injury (SCI) now recognized as the predominant underlying etiology [4-8].

The pathophysiology of SCA is multifactorial. One proposed mechanism involves autonomic dysfunction following neurological injury, leading to increased bone resorption in the hypervascular subchondral bone, resulting in microfractures, progressive joint destruction, and vertebral instability [9,10]. Another theory centers on the biomechanical consequences of proprioceptive loss in SCI, which compromise normal stabilizing mechanisms. This results in excessive biomechanical loading, joint capsule hypertrophy, and progressive vertebral instability and subdislocation. This process may be of particular relevance among paraplegic patients who compensate for their altered functional status with movements that place repetitive biomechanical load on the spine, such as self-transfers [11]. Notably, SCA may develop at the level or adjacent levels to the site of initial SCI. Prior spinal interventions have also been associated with increased risk of disease at the surgical site [12-15].

SCA often presents insidiously, with subtle, nonspecific symptoms that may include paroxysmal or constant back pain, spinal instability and deformity, audible “clicking” sensations, changes in neurological function, or new-onset autonomic dysreflexia (AD). Onset typically occurs decades after the initial neurologic insult, with most cases reported in the fifth decade of life [4,7,9]. Due to its subtle onset and diagnostic challenges, SCA is frequently underrecognized until substantial spinal compromise has occurred. Despite the complex and progressive nature of this condition, current guidelines for diagnosis, treatment, and long-term management remain limited.

In this report, we present the case of a 28-year-old female patient with a severe presentation of SCA at a relatively young age. We detail the diagnostic process, surgical considerations, and postoperative management to contribute additional evidence to the limited body of literature, which may inform future care of patients with SCA.

## Case presentation

Patient overview

A 28-year-old female patient with a past medical history of paraplegia secondary to Asia A T5 SCI presented to our clinic with 1.5 years of worsening posterior thoracic prominence in the setting of a Charcot joint of the thoracic spine with fracture translocation of T12 associated with a pseudomeningocele (Figures 1-3). Her initial SCI had occurred over a decade prior and was related to a complicated spinal cord tethering for which surgical correction had been attempted. The patient had been referred from out of state, and complete details regarding the index injury and prior operative intervention were unavailable despite review of the transferred records.

**Figure 1:**
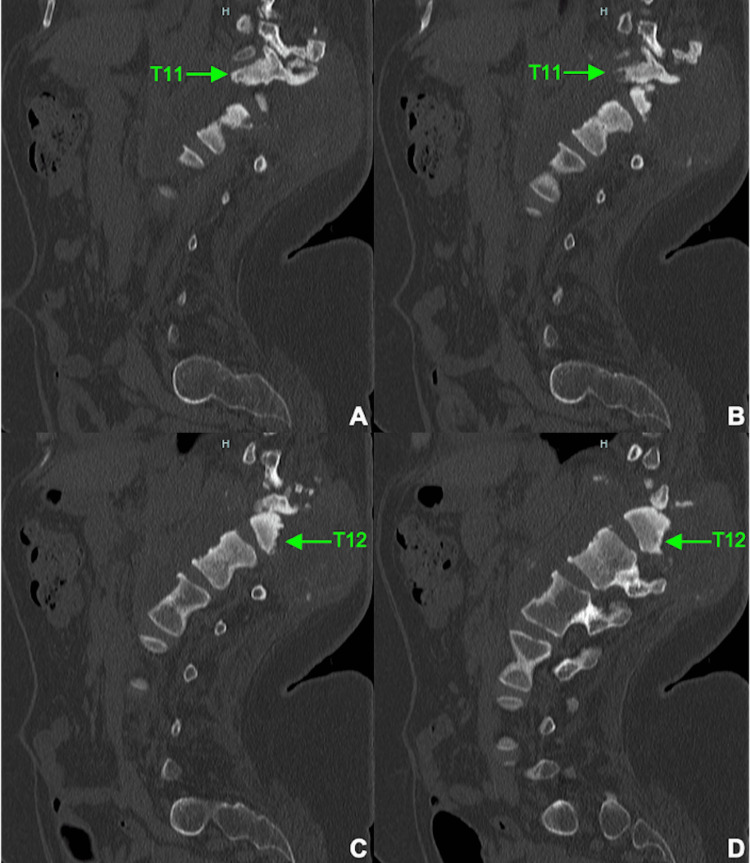
Sagittal CT images (A-D) demonstrate chronic transection of the thoracic spinal cord at the T11-T12 level with an associated neuropathic joint and surrounding pseudomeningocele. There is posterior and leftward dislocation of T12 relative to T11, along with erosive changes involving the T12-L2 vertebral bodies. Arrows in (A) and (B) indicate the T11 vertebral body, while the arrows in (C) and (D) highlight the T12 vertebral body at the site of dislocation and osseous destruction

**Figure 2:**
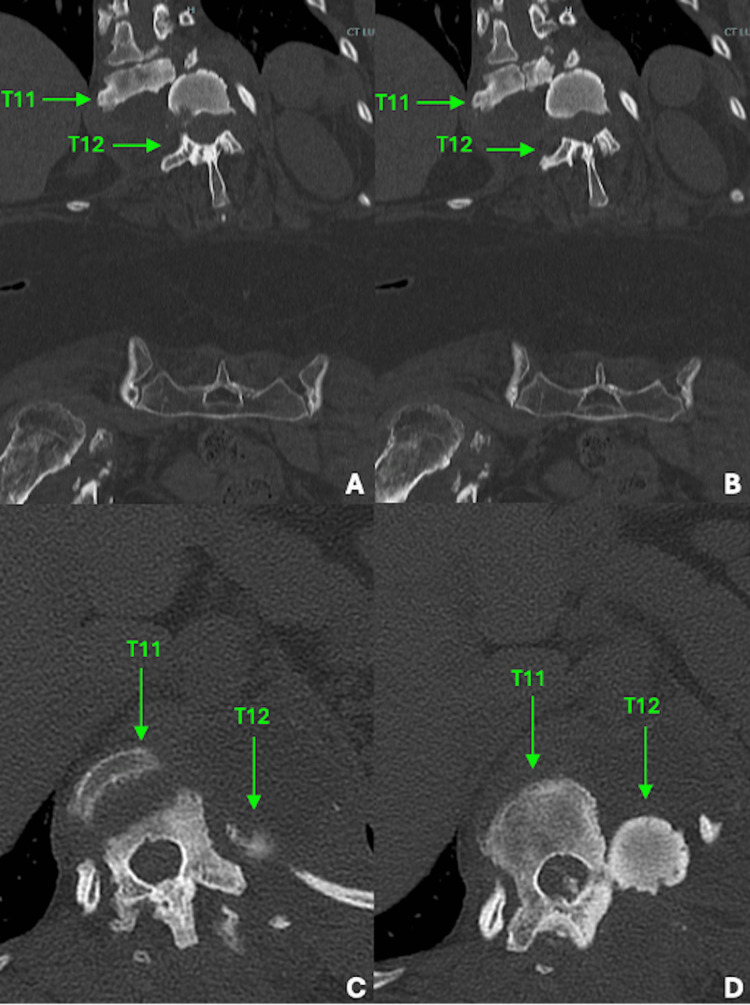
Coronal CT views (A,B) demonstrate posterior and leftward dislocation of T12 relative to T11, with associated erosive changes involving the T12-L2 vertebral bodies. Axial CT views (C,D) demonstrate chronic erosive changes and pseudomeningocele formation adjacent to the site of spinal cord transection. Arrows in (A-D) indicate the T11 and T12 vertebral bodies, highlighting their malalignment and abnormal anatomy at the level of injury

**Figure 3:**
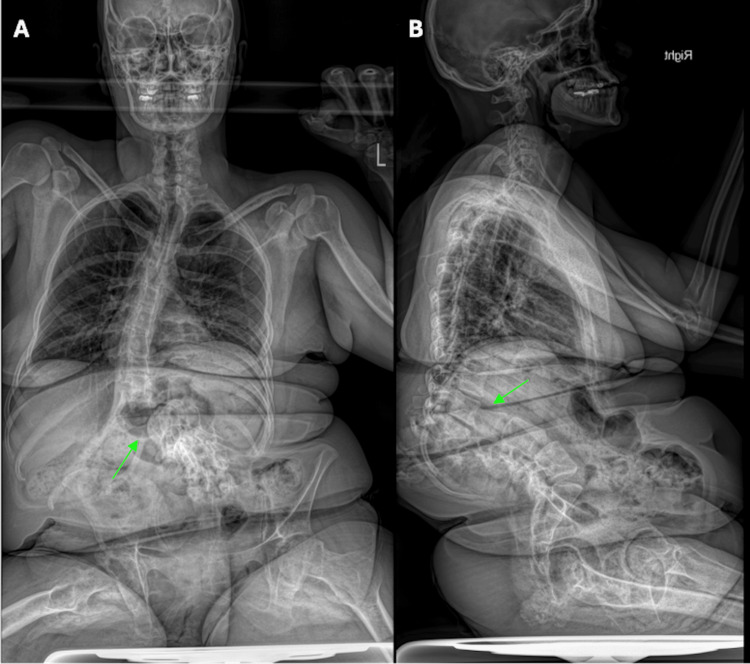
(A) AP and (B) lateral views demonstrate a severe thoracolumbar deformity with posterior and leftward dislocation of T12 relative to T11 (arrows), along with collapse and erosive changes involving the T12-L2 vertebral bodies, consistent with a neuropathic (Charcot) spinal joint. These findings correspond to the chronic spinal cord transection at the T11-T12 level and associated pseudomeningocele formation identified on preoperative CT imaging AP: anteroposterior

Upon presentation, she noted that this thoracic prominence had worsened over time and now impaired her ability to sit up and function in her wheelchair. This deformity was grossly reducible with transfers from the bed to her wheelchair. Her neurologic exam was consistent with her baseline T5 SCI with absence of motor function and sensation in the bilateral lower extremities, but intact motor and sensory function in the bilateral upper extremities. Even with the development of Charcot joint and thecal sac injury, she had reported no worsening in neurological symptoms or positional headaches. A surgical plan of T5 to pelvis fixation was presented to the patient, who ultimately provided informed consent.

Intraoperative procedure

The patient was placed prone on an open Jackson table in Mayfield pins with pressure points appropriately padded. Head immobilization was used to optimize prone positioning, maintain global spinal alignment during deformity reduction, and minimize unintended motion during vertebrectomy and reduction maneuvers. Bone marrow aspiration from the pelvis was performed prior to incision, and the exposure of T4 through the sacrum was performed in standard fashion. Exposure of T4 was performed to facilitate accurate identification of the T5 level, allow safe placement of proximal instrumentation, and optimize rod contouring and construct alignment. No instrumentation was placed at T4. Fusion was terminated at T5 to anchor the construct into stable bone above the denervated and biomechanically compromised thoracolumbar spine. Imaging did not demonstrate Charcot changes, fracture, or instability at or above T5. Extending fusion more proximally was felt to increase morbidity without additional biomechanical benefit. Immediately upon exposure, a free-floating severely disrupted vertebral body was encountered. The spinal cord and thecal sac were completely transected with an associated pseudomeningocele, which was opened and drained on initial exposure (Figure 4).

**Figure 4:**
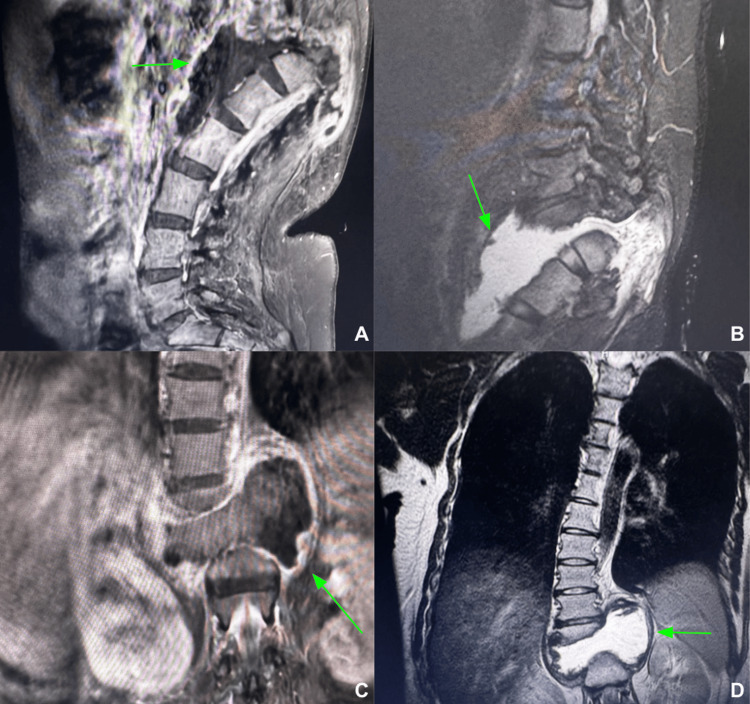
Preoperative sagittal and coronal MRI images. Sagittal MRI views demonstrate the thoracolumbar deformity and associated pseudomeningocele at the level of chronic spinal cord transection: (A) T1-weighted imaging and (B) T2-weighted imaging. Coronal MRI views: (C) T1-weighted imaging and (D) T2-weighted imaging. The images demonstrate extensive pseudomeningocele formation adjacent to the T11-T12 level, associated with chronic spinal cord disruption and surrounding deformity. Green arrows in (A-D) identify the pseudomeningocele and abnormal anatomy at the level of injury

Intraoperative neuromonitoring was utilized throughout the procedure in accordance with standard institutional practice for complex spinal deformity reconstruction. Medtronic navigation was used after an intraoperative O-arm spin. Prior to T12 vertebrectomy and reduction, segmental instrumentation was placed throughout the thoracic and lumbar spine using a navigated high-speed burr and awl-tip tap. This allowed for cannulation of a trajectory that could be used for bilateral pedicle screws. Additionally, bilateral iliac bolts were placed, along with S1 and S2 alar-iliac trajectories to achieve pelvic and sacroiliac joint fixation. Confirmation of adequate placement and absence of breached instrumentation was confirmed through intraoperative CT imaging.

After screw placement, attention was turned to the T12 vertebrectomy. A complete resection of the displaced T12 vertebral body was performed, including bilateral lamina, spinous processes, pedicles, transverse processes, and the vertebral corpus.

Spinal realignment was initiated after complete vertebrectomy. Temporary rods were placed prior to vertebrectomy for provisional stabilization and were subsequently repositioned during deformity reduction. Temporary rods were placed on the left and used for manual reduction with rod holders. A right-sided temporary rod was then placed to maintain reduction, while a precut, precontoured 5.5-mm titanium S-rod was positioned on the left and sequentially reduced into the tulip heads for final fixation. The procedure was repeated on the right side with a second prebent S-rod. An additional J-rod was placed bilaterally, spanning from the iliac bolts to the level of the fracture. The J-rod was then secured to the iliac bolts and the main rods using side connectors. Intraoperative serial X-rays were used to confirm the extent of reduction and proper maintenance of alignment.

Attention was then directed toward the trauma-associated pseudomeningocele, which likely resulted from the patient’s fracture/dislocation, which had transected the spinal cord and thecal sac, allowing persistent cerebrospinal fluid (CSF) leak from the lacerated dura. The pseudomeningocele was opened and drained during exposure. Because the spinal cord had no remaining function at the level of the operation and primary dural repair was inconceivable due to the extent of her injury, we elected to tie off the thecal sac and cord to address the CSF leak. A complete laminectomy from T9-T11 was performed. The thecal sac was dissected circumferentially, and three 0-silk ties were tied with enough tension to tighten the dura against the underlying cord, preventing CSF flow. Duragen (Integra LifeSciences, Princeton, NJ) and Adheurs (Stryker, Portage, MI) were also placed on both the proximal and distal ends of the transected thecal sac.

An expandable corpectomy cage was placed into the T12 defect and expanded to the desired height. Appropriate positioning of the cage was confirmed with AP and lateral fluoroscopy. To promote proper spinal fusion, the expandable cage was packed with bone morphogenetic protein and morselized autograft and covered in a strip of dural sealant.

Posterior arthrodesis was then performed from T5 to S2. The facet joints were decorticated and filled with bone morphogenetic protein and morselized autograft and sealed with a dural sealant. Additionally, bioactive glass graft substitute strips that had been soaking in platelet-rich plasma, as well as P-15 peptide-enhanced bone graft substitute, were placed in the posterolateral gutters. Using this process, arthrodesis was performed from T5-S1 and across S1-S2 for iliac fixation. The wound was closed in the usual manner. An incisional wound vacuum-assisted closure was applied. There were no intraoperative complications.

Postoperative course

Following surgery, the patient was admitted to the intensive care unit. She reported no pain, just some nausea and dizziness. It was noted that the patient was hypotensive with systolic pressures in the 60s to 90s, most likely due to a multifactorial autonomic dysfunction. Midodrine was administered with adequate results. Discharge arrangements were made for an acute rehab center. In total, the patient had an eight-day postoperative course in the hospital with minor complications. At a two-week follow-up, her incision was overall well-healed. The patient did develop a urinary tract infection at the acute rehab center, likely secondary to an indwelling catheter. At the three-month follow-up, her EOS (EOS Imaging, Paris, France) X-rays were obtained (Figure 5).

**Figure 5:**
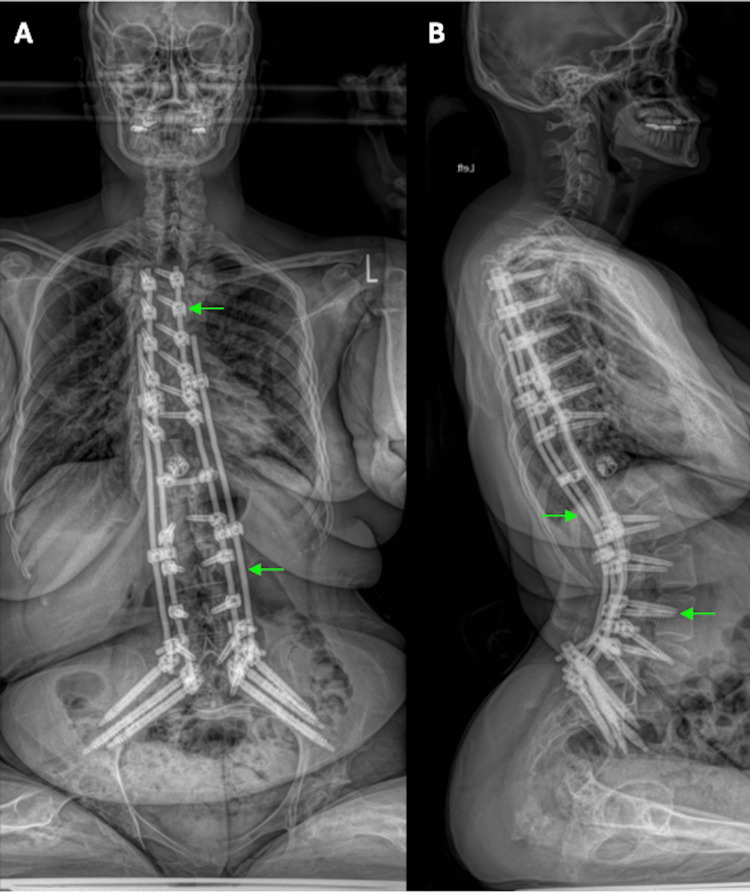
(A) Anteroposterior and (B) lateral views demonstrating status post posterior spinal fusion from T5 to S2 with transpedicular screws and rods (arrows) in satisfactory position and overall alignment. Although visualization at the operative levels is limited on plain radiographs, there is a marked reduction in thoracolumbar subluxation compared with preoperative CT imaging

## Discussion

We have described the clinical course of a successful T5 to pelvis fixation in a young female patient with SCA. Given the complex nature of this case, several important factors were discussed and considered in the preoperative surgical planning of this case.

Fusion length

Determining the appropriate fusion length in SCA is critical to ensuring long-term spinal stability and preventing adjacent segment disease. Prior studies have demonstrated that limiting fusion to the lumbar spine may increase the risk of developing a secondary Charcot joint, likely due to residual biomechanical stress on adjacent mobile segments [14]. Moreover, when the fusion is limited to the denervated segment of the spine, there is a higher rate of hardware failure and development of SCA at adjacent segments. In contrast, constructs extending beyond the denervated spine to healthy bone, such as the pelvis, have shown higher overall fusion rates and lower incidences of disease recurrence [16]. In this case, a long-segment fusion to the pelvis was performed to maximize construct durability and minimize future instability.

Posterior vs. circumferential fusion

The optimal approach to fusion, posterior-only vs. circumferential, remains an area of debate, particularly in SCA, where bone quality is often poor and biomechanical forces are complex. Posterior-only constructs are associated with higher hardware failure rates, especially at the lumbosacral junction, where shear forces are considerable [11,14,17]. Circumferential (anterior-posterior) fusion offers enhanced anterior column support and has been associated with improved construct longevity and reduced rates of secondary Charcot joint formation and revision surgery [13]. Additionally, some studies have reported improved neurological outcomes following circumferential fusion, including return to baseline function [15]. Despite these advantages, revision rates remain high across both approaches, underscoring the technical challenges and progressive nature of SCA. Principles used in the management of other destructive spinal osseous lesions, with an emphasis on circumferential stability and durable biomechanical fixation, may similarly apply to reconstruction in advanced SCA.

Surgical staging

The decision between single-stage and staged circumferential fusion is guided by the patient’s overall medical and surgical status. In patients who are hemodynamically stable and surgically optimized, a single-stage approach is preferred to reduce operative burden and complications [6,18-20]. When a single-stage procedure is contraindicated, a staged approach may be used, though it has been associated with increased morbidity and mortality. Careful preoperative assessment is essential to selecting the safest and most effective surgical strategy.

Surgical instrumentation

Given the high rates of hardware failure in SCA, careful consideration must be given to construct the design. While traditional two-rod constructs are widely used, multirod techniques, particularly four-rod constructs, have gained support for improving stability across multiple planes, increasing stiffness, and reducing micromotion at the lumbopelvic junction [21-23]. These benefits are relevant in long-segment fusions, where forces across the construct are magnified. Additionally, patients with SCA are at an increased risk of hardware failure and revision in long-term follow-up studies using standard two-rod constructs [11,14]. In this case, a four-rod construct was used to enhance biomechanical stability and mitigate the risk of failure. Prior reports have shown that this approach is associated with reduced instrumentation failure and improved clinical outcomes, including resolution of associated symptoms such as AD [14,24-26].

Management of thecal sac injury

A distinctive feature in this patient was the presence of a pseudomeningocele on presentation, suggesting thecal sac violation as a result of progressive osseous destruction. Interestingly, the patient’s baseline neurological function remained unchanged. While the literature rarely addresses thecal sac involvement in SCA, one prior case described management of a CSF-cutaneous fistula via dural ligation and transection [27]. To our knowledge, this is the first report of a pseudomeningocele identified on initial imaging in a patient with SCA. Due to the extent of tissue destruction and complete loss of distal neurologic function, primary repair of the thecal sac was not feasible intraoperatively. Therefore, definitive ligation of the transected thecal sac was performed in an effort to control persistent CSF leakage. Adjunctive dural sealants and collagen matrix grafts were additionally applied to reinforce the closure, both of which have demonstrated efficacy in reducing postoperative CSF leak rates [28,29]. The successful management in this case highlights the importance of identifying and definitively addressing thecal sac compromise in patients with advanced SCA.

AD as a comorbidity

AD is a known complication of both SCI and SCA and may be precipitated by surgical stress or inadequate pain control [26,30]. It is believed to result from impaired supraspinal modulation of sympathetic outflow, leading to unregulated vasoconstriction and potentially life-threatening hypertension. Triggers include bowel or bladder distention, infection, pain, or positioning below the level of injury [30-33]. Prevention involves comprehensive perioperative management, including analgesia, bowel/bladder regulation, and minimization of noxious stimuli [30,34]. Intraoperative and postoperative monitoring of vital signs, particularly blood pressure, is essential [34,35].

Differential diagnosis and imaging considerations

Although alternative etiologies such as progressive deformity secondary to prior destabilizing decompression must be considered, the extensive osseous destruction, pseudomeningocele formation, fracture-dislocation, and progressive instability observed in this case were felt to be most consistent with SCA. Furthermore, prior literature supports the development of Charcot arthropathy both adjacent to and remote from the original level of SCI, particularly in patients with long-standing denervation and altered spinal biomechanics following prior spinal intervention [11-16].

In paraplegic patients presenting with progressive deformity or functional decline, differential considerations may additionally include plexopathy, infection, hardware failure, and progressive deformity secondary to prior spinal intervention. Cross-sectional imaging with CT and MRI plays an important role in distinguishing these entities and characterizing osseous destruction, instability, pseudomeningocele formation, and neural element involvement.

## Conclusions

This case highlights the importance of maintaining a high index of suspicion for SCA even in young paraplegic patients presenting with new or subtle symptoms such as back pain, spinal deformity, or changes in neurological or autonomic function. Early recognition remains challenging but is essential, as delayed diagnosis can lead to progressive deformity, instability, and neurological compromise. Given the absence of standardized diagnostic or management guidelines, a multidisciplinary approach is critical.

Our case underscores the utility of individualized surgical strategies, including anterior column support, definitive dural management, and quad-rod constructs, in achieving stable spinal reconstruction and potentially minimizing recurrence. Furthermore, continued clinical and radiographic surveillance is imperative, as multifocal or recurrent disease is not uncommon in this population. This case also highlights the importance of considering SCA among the differential diagnoses for progressive deformity in chronically paraplegic patients, while also recognizing alternative etiologies such as prior destabilizing surgery. This report contributes to the growing body of literature on Charcot spine in young patients and may help guide future treatment approaches.
